# Evaluation of Maxillary Molar Distalization Supported by Mini-Implants with the Advanced Molar Distalization Appliance (amda^®^): Preliminary Results of a Prospective Clinical Trial

**DOI:** 10.3390/jcm14176323

**Published:** 2025-09-07

**Authors:** Nikolaos Karvelas, Aikaterini Samandara, Bogdan Radu Dragomir, Alice Chehab, Tinela Panaite, Cristian Romanec, Moschos A. Papadopoulos, Irina Nicoleta Zetu

**Affiliations:** 1 Department of Orthodontics, Faculty of Dentistry, University of Medicine and Pharmacy “Grigore T. Popa”, 700115 Iasi, Romania; karvelas93@gmail.com (N.K.); dragomirbo@gmail.com (B.R.D.); alicechehab1992@gmail.com (A.C.); tinelap@yahoo.com (T.P.); nicoleta.zetu@gmail.com (I.N.Z.); 2 Department of Orthodontics, Faculty of Dentistry, School of Health Sciences, Aristotle University of Thessaloniki, 54124 Thessaloniki, Greece; katerinasamandara@gmail.com (A.S.); mikepap@dent.auth.gr (M.A.P.)

**Keywords:** distalization, amda^®^, mini-implants, Class II malocclusion, non-compliance appliances

## Abstract

**Background:** Class II is considered one of the most common malocclusions, influencing 37% of schoolchildren in Europe and 33% of orthodontic patients in the United States. When this type of malocclusion is combined with increased overjet with proclined teeth and maxillary excess, then moving maxillary molars distally is suggested. According to the recent literature, modern appliances that lack patient cooperation can be combined with temporary anchorage devices to provide absolute and skeletal anchorage while supporting the non-compliance appliances to eliminate their side effects, such as anterior and posterior anchorage loss along with maxillary molar inclination and rotation. To counteract these limitations, the Advanced Molar Distalization Appliance (amda^®^), a non-compliance appliance for maxillary molar distalization supported by two mini-implants (MIs) with anterior abutments, was recently developed. **Methods:** In this preliminary prospective clinical trial, eight consecutive patients treated with the amda^®^ are evaluated through lateral cephalometric radiographs, while its application, construction, and anchorage is presented and discussed. The evaluation of dentoalveolar and skeletal changes has been made with 14 variables measured on the pre- and post-cephalometric radiographs before and immediately after maxillary molar distalization (T_0_ and T_1_, respectively), along with cephalometric superimpositions by the structural method. **Results:** In total, the mean distal molar movement was 4.2 ± 1.37 mm, the mean distal tipping was 1.7 ± 1.9 degrees, and the vertical movement was 1.6 ± 2.6 mm. **Conclusions:** The amda^®^ seems to provide an ideal option for treating patients with Class II malocclusion, achieving bodily movement of the maxillary molars with only minimal distal tipping and no anchorage loss.

## 1. Introduction

One of the most common malocclusions is a dental Class II occlusion, influencing 37% of schoolchildren in Europe and 33% of orthodontic patients in the United States [1]. When this type of malocclusion is combined with increased overjet with proclined teeth and maxillary excess (maxillary prognathism), the moving of the maxillary molars distally is usually suggested; however, the choice of the treatment may vary depending on the specialist’s clinical judgment and the healthcare system in which the patient is treated. Many patients consider it unpleasant to wear extraoral headgear due to its esthetic appearance and treatment duration limitations, and are usually non-compliant. Therefore, intraoral non-compliance appliances were introduced for patients with limited cooperation [2,3,4,5].

In addition, the proportion of patients undergoing extractions as part of orthodontic treatment has significantly declined in recent years, since multiple studies have demonstrated that extractions do not always ensure outcome stability [3]. Thus, the non-extraction protocols have been implemented more and more frequently. Various approaches have been introduced either with conventional mechanics or/and non-compliance appliances in order to achieve Class I molar relationships [4,5,6]. According to the recent literature, modern appliances that do not need patient cooperation along with the use of temporary anchorage devices (TADs) seem to provide a better solution for maxillary molar distalization and can withstand the reciprocal forces that may create a number of side effects [7,8]. TADs can provide absolute and skeletal anchorage while supporting the non-compliance appliances eliminating side effects, such as anterior and posterior anchorage loss along with maxillary molar distal tipping and bucco/lingual rotation. Several clinical trials have investigated the effectiveness of various orthodontic devices for the distalization of molars with TADs. These studies typically assess devices such as pendulum appliances, distal jet appliances, and modified anchorage devices. Trials often compare outcomes like treatment duration, amount of molar movement, anchorage loss, and patient comfort. For instance, randomized controlled trials have shown that mini-screw-anchored systems tend to offer greater distal movement with better anchorage control and less tipping compared to traditional tooth-supported appliances [9,10,11].

To overcome these limitations, the Advanced Molar Distalization Appliance (amda^®^), a non-compliance appliance for maxillary molar distalization supported by two mini-implants (MIs) with anterior abutments, was recently developed [12,13,14,15].

Until now, there has been no clinical trial that has evaluated its effectiveness in patients with permanent dentition.

The purpose of this prospective study was to evaluate the efficiency and effectiveness of the amda^®^ for maxillary molar distalization.

## 2. Materials and Methods

This article described a prospective clinical trial of eight consecutive patients—four males and four females—who were treated with the amda^®^. The patients, aged between 14 and 20 years, received treatment at the Department of Orthodontics, of the University of Medicine and Pharmacy “Grigore T. Popa”, in Iasi, Romania. All patients were thoroughly documented including panoramic radiographs, cephalometric radiographs, CBCT scan of the maxilla, and digital 3D models as well as intraoral and extraoral photographs. Cephalometric radiographs were collected for assessment at the beginning of the treatment at the initial stage (T_o_) and at the end of the distalization treatment at the final stage (T_1_).

This study was approved by the Ethical Committee of the UMF, Iasi (**Nr. 233/11.11.2022**) and is registered on ClinicalTrials.gov with the registration number NCT06011382, and a registration date of 24/08/2023. In addition, an informed consent was obtained from the patients or the parents of the patients, along with a patient’s information form. Patients were free to drop out at any stage of the treatment.

In this study, patients were included with the following criteria: All patients must have permanent dentition with or without the second molar erupted and bilateral Class II molar relationships (from a quarter to one molar cusp). On the contrary, patients with past orthodontic treatment, crossbites, severe carious lesions, poor oral hygiene, mobility of the maxillary first molars, anterior open bites, vertical growth pattern, and tongue habits were excluded from the current investigation.

### 2.1. Clinical Application of amda^®^

**Figure 1:**
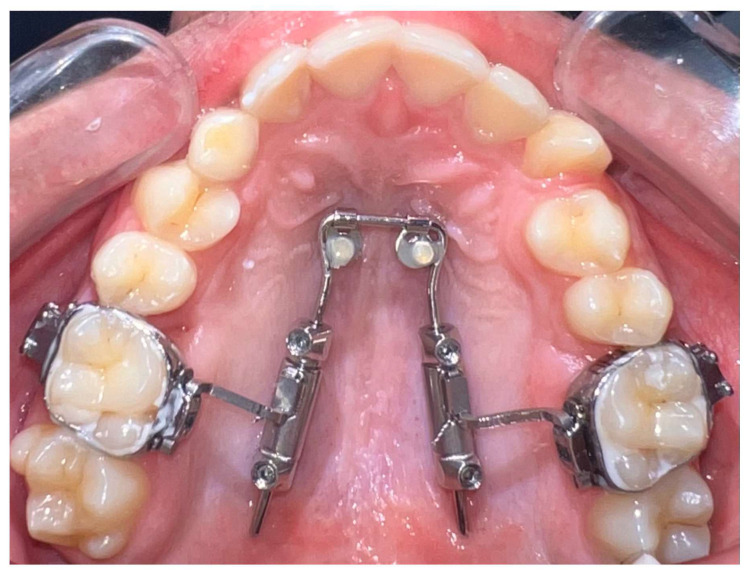
The amda^®^ on two mini-implants (2.0 × 10 mm; Tomas Pin EP; Dentaurum, Germany).

The amda^®^ is a prefabricated device anchored to the palate by two mini-implants and it is a non-compliance appliance for maxillary molar distalization (Figure 1).

The aim behind developing amda^®^ is to initially achieve a pure bodily distal movement of maxillary molars without anchorage loss of the anterior teeth, and, later, during the retraction of the anterior teeth to avoid posterior anchorage loss through the mesial movement of the molars that have been previously distalized [14].

It comprises (1) a tubing system with encased compressed nickel–titanium (Ni-Ti) open coil springs to provide the necessary distalization force, (2) a horseshoe-type palatal archwire with two movable abutments, on which the tubing system slides, (3) a palatal anchorage unit consisting of two mini-implants with paramedian positioning [12,13,14,15], and (4) conventional orthodontic bands on maxillary first molars equipped with lingual sheaths.

**Figure 2:**
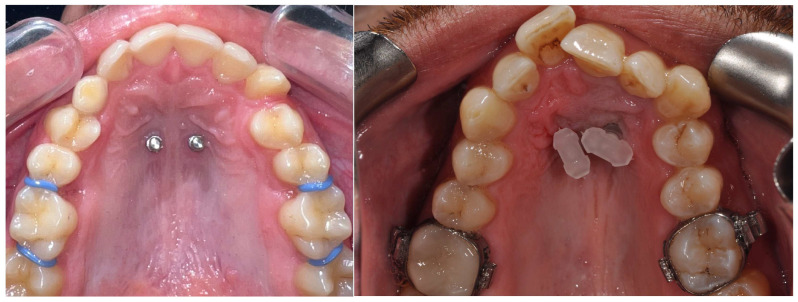
Two mini-implants were placed in the paramedian region. Transferred caps placed on the two mini-implant heads.

To insert the appliance in the mouth, following placement of the MIs, bands are cemented on the molars and the special transferred caps are placed on the head of the MIs (Figure 2).

**Figure 3:**
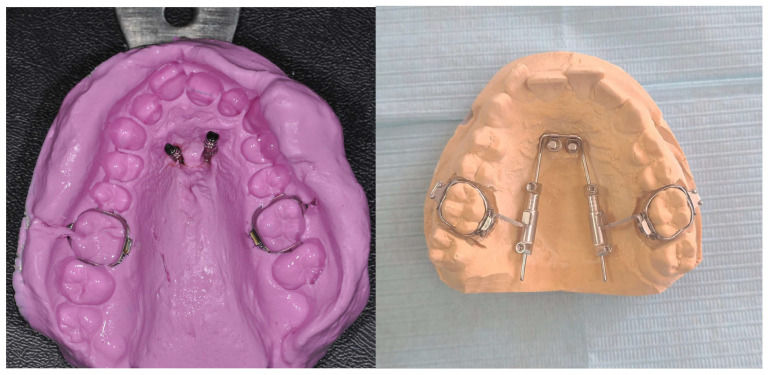
Mini-implant analogs were placed into the alginate impression to deliver to the laboratory. The amda^®^ in a plaster cast model with bands and lab analogs adjusted.

Then, an alginate or polyvylisiloxane impression is taken; the mini-implant analogs are put into the impression and transferred to the laboratory for cast model pouring and the subsequent adaptation of the prefabricated appliance (Figure 3).

After the appliance is adapted on the model cast, it is inserted in the palatal sheaths of the molar bands in the mouth, and then the abutments are connected to the MI heads. Finally, after initial activation of the amda^®^ by pushing and screwing the mesial screw and by unscrewing the distal screw on each tubing system with the special hexagonal key, reactivations follow every 4–6 weeks.

### 2.2. Anchorage Unit of the amda^®^

**Figure 4:**
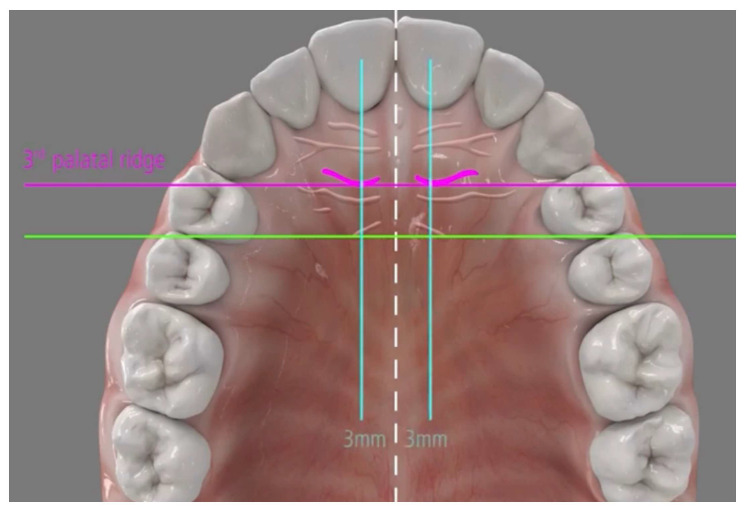
Placement of two mini-implants through the gingival tissue in the paramedian region of the palate, 3–6 mm from the midpalatal suture and 3–6 mm posterior to the incisive foramen. Figure taken with permission from Papadopoulos, M.A. (Ed.) *Skeletal Anchorage in Orthodontic Treatment of Class II Malocclusion*; Elsevier, Mosby: Edinburgh, UK, 2015 [14].

Placement of two mini-implants (Tomas Pin EP; Dentaurum, Germany), with 2 mm diameter and 8–10 mm length, are inserted directly through the gingival tissue or the mucosa without any need for pre-drilling in the paramedian region of the palate 3–6 mm from the midpalatal suture and 3–6 mm posterior to the incisive foramen (Figure 4).

The mini-implant insertion is achieved either manually with the use of screwdrivers or a rachet or mechanically with a specially angled hand-piece, with a speed up to 30 rpm (which is usually adequate and should not exceed 60 rpm), while the ideal force level of the screwdriver must be 10 Ncm.

In the current clinical investigation, the mini-implants were placed by an experienced oral surgeon while the amda^®^ was adapted and inserted by a junior orthodontist specialist. Pre- and post-cephalometric radiographs and 3D models were collected for analysis at the beginning (T_o_) and at the end of the distalization (T_1_) in a period from 10 to 12 months. However, this preliminary article will present only the cephalometric variables, while all the outcomes of this clinical trial will be presented in a future article when the study is completed.

### 2.3. Evaluation of the Distal Movements and Its Adverse Effect

Evaluation of dentoalveolar and skeletal changes was made with 14 cephalometric variables as follows:

- 3 angular measurements were used to assess skeletal changes;
- 5 angular and 6 linear variables were included to assess dentoalveolar measurements (Table 1).

Cephalometric variables are presented in Table 1. Dentoalevolar angular and linear measurements were measured and compared to evaluate the initial and final position of the maxillary molars during the distalization phase, providing indications of both bodily movement and tipping.

**Figure 5:**
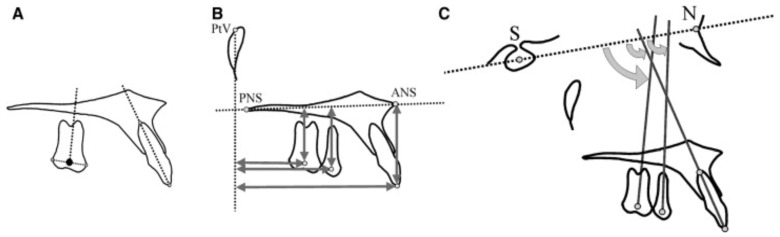
(**A**) The constructed centroid point of the maxillary first molar. (**B**) Cephalometric dentoalveolar linear measurements. (**C**) Cephalometric dentoalveolar angular measurements. Figure taken with permission from Papadopoulos MA, Melkos AB, Athanasiou AE [6].

A point at the center of the posterior teeth (centroid) was used to determine their actual position in the sagittal and the vertical plane (Figure 5).

This point represented the midpoint between the greatest mesial and distal convexity of the crowns as seen on the cephalometric radiograph. To assess the dental changes in the horizontal and sagittal planes, measurements were made from the centroid points of the pterygoid vertical (PTV) and palatal (ANS-PNS) reference planes, respectively.

The long axes of the premolars and molars were constructed by drawing a perpendicular from the centroid to a line connecting the most convex points of the crowns of those teeth. Angular changes were estimated concerning the inclination of the long axes to the sella–nasion (SN) plane. In case of double projection of the molars or premolars, a medial contour has to be traced and used for the corresponding measurements.

Pre- and post-cephalometric radiographs were collected and evaluated digitally before T_o_ and after the distalization period T_1_ using the WebCeph analysis software (version 1.0.3, WEBCEPH, Pangyoyeok-ro, Bundang-gu, Seongnam-si, Gyeonggi-do, Republic of Korea). A total of eight pre- and eight post-cephalometric radiographs were evaluated. Then, in every case, a superimposition of the initial and final cephalometry was undertaken to confirm the outcomes.

Cephalometric digital superimposition was evaluated by the structural method of profile radiographs in the anterior cranial base based on the observation by the Björk structural method (M1): radiographs were superimposed on the reference bone structures in the anterior cranial base, as described by Björk and Skieller [16]. Tracing the nasion–sella line (NSL) and a perpendicular line through the sella point (NSP) were marked directly on the first radiograph. The subsequent radiographs were superimposed according to the structures on the first radiograph. The sella point and the crosslines were then transferred from the first to the subsequent radiographs.

To assess the error of the method, cephalometric measurements and superimpositions were double-evaluated and undertaken independently by two authors (N.K., A.S.), while a third author settled any disparities (M.A.P.).

### 2.4. Statistical Analyses

The differences in consecutive measurements between the two timepoints for each patient were calculated, and descriptive statistics (mean and standard deviation) were computed. The normality of these differences was assessed using the Shapiro–Wilk test. Paired *t*-tests and Wilcoxon signed-rank tests were applied to evaluate on pre- and post-treatment cephalometric radiographs, as appropriate. Bonferroni corrections were implemented to adjust for multiple comparisons, where appropriate. Differences were considered statistically significant at *p* < 0.05, unless adjustments for multiple comparisons were implemented. All statistical analyses were performed using R/R-Studio version 2024.04.2 + 764 (Posit Software, PBC).

### 2.5. Evaluation of the Error of the Method

Intra-examiner reliability was assessed by re-evaluating the cephalometric measurements at timepoints T_0_ and T_1_. These measurements were retraced, digitized, and remeasured 40 days after the initial assessment by two different authors. The differences between the initial and subsequent measurements were analyzed, and the random (casual) error was calculated using Dahlberg’s formula, where “d” denotes the difference between the first and second measurements and “n” refers to the number of repeated cases. In addition, the systematic error was evaluated using a paired *t*-test in accordance with the method proposed by Houston. A significance level of *p* < 0.05 was adopted for all statistical analyses.

## 3. Results

The random error, calculated using Dahlberg’s formula, across all cephalometric variables indicated the acceptable reproducibility of the measurements. The systematic error that was assessed using paired *t*-tests revealed that no statistically significant differences were found between the initial and repeated measurements (*p* > 0.05 for all variables), suggesting the absence of consistent measurement bias.

The distal molar movement continued until a Class I relationship was achieved bilaterally for each patient. Mean distalization time was calculated between 10 and 12 months. The mean distal molar movement was 4.2 ± 1.37 mm, indicating bodily movement as measured by the PTV-6 variable. The mean distal tipping was 1.7 ± 1.9 degrees based on the Palatal Plane-6 parameter, while the vertical movement averaged 1.6 ± 2.6 mm, as indicated by the SN-6 measurement (Table 2).

Although the sample size was limited, the statistical analysis revealed a significant change in the PTV-1 (mm) dentoalveolar linear measurement, indicating a sagittal movement of the maxillary central incisors despite the absence of any applied force. In addition, a statistically significant difference was observed in the PTV-6 in the distal molar movement measurements across the different timepoints (Table 2 and Table S1).

The amount of positional changes was calculated by the difference between pre- and post-superimposition cephalometric measurements with Bonferroni test and paired *t*-test to assess the amount of displacement over T_0_ and T_1_ differences. The outcomes of Björk structural method demonstrated no statistically significant difference (*p* > 0.05) among the different timepoints (Table 3).

Each patient’s initial and final lateral radiography was precisely calculated, and all measurements were taken from the lateral cephalograms. However, the mean rotation and transverse expansion were not possible to be measured, as only lateral cephalograms were included in this study.

## 4. Discussion

Unlike traditional non-compliance devices, during molar distalization, amda^®^ creates spaces between the maxillary first molars and second premolars, as well as between the second premolars and first premolars, while simultaneously generating spaces between all the anterior teeth. This is due to the fact that the premolars and canines are able to drift distally under the pull of the transeptal fibers, which also shortens overall treatment time [17]. Usually, a minimum of three months is required for space to become noticeable. The undesirable complications of conventional intraoral devices are avoided by its unique design and the employment of palatal mini-implants, which provide temporary and stationary anchorage for all treatment stages [18]. The anchorage unit consists of two self-drilling and self-tapping mini-implants to resist the reciprocal forces during molar distalization and, later, during anterior teeth retraction, while the ideal site of the MIs is the paramedian region [17,18,19,20]. The insertion of the two MIs in the region behind the incisive foramen provides a safety clearance of 7–10 mm between the MIs and the dental roots of the anterior teeth. Thus, this insertion site avoids contact of the MIs and these teeth during molar distalization and, more importantly, during anterior teeth retraction [12,14]. The MIs are recommended to be inserted parallel to each other. In any case, although it is not required, a surgical guide can be used for more precise placement of the MIs.

In the current study, in two of our patients, the amda^®^ had to be removed for 2 weeks due to penetration of the palatal archwire into the palatal mucosa. The reason for this was the rotated upper molars. Interestingly, despite the archwire perforating the mucosa and traumatizing the palate, the patients experienced no discomfort or pain, which may be attributed to the hard porous nature of the palatal vault. After complete healing, distalization continued with an active bend to adjust the force direction. In addition, one patient dropped out of the study for personal issues.

Regarding the MIs in our study, only 2 out of 16 MIs failed during the insertion procedure. However, after placement, none failed, supporting previous studies that identify the palate region as the safest and most reliable site for anchorage.

According to the findings of this investigation, the amda^®^ was able to distalize the first maxillary molars efficiently by approximately 4.2 mm on average, with a minimal molar tipping of 1.7 degrees and 1.6 mm of vertical movement. It should be noted, however, that, in one case, the highest recorded distal molar movement was 6 mm. It is important to highlight that all eight cases were treated over different monthly time periods, as each patient required a customized timeline to achieve a Class I molar relationship. This variation can be explained by the differing types of malocclusions presented, ranging from half-cusp to full-cusp distal relationships. The observed minimal molar tipping (1.7°) was significantly lower compared to findings from other studies with similar approaches [21]. Additionally, a statistically significant difference was observed in the PTV-6, the PTV-1, and in the overbite measurements across the different timepoints.

Initial and final lateral cephalometric radiographs after molar distalization were used to assess the bodily movement and tipping of the first maxillary molars along with vertical measurements. Although these radiographs are superior and more effective for measuring molar inclination and vertical movements, transverse movements and rotations cannot be evaluated accurately; for these measurements, 3D evaluation on model casts is suggested.

Although this study lacked a control group, it is unlikely that the non-growing Class II patients would have experienced distal displacement of the maxillary molars on their own. Moreover, it is considered unethical to withhold treatment from a patient diagnosed with orthodontic issues that require intervention. Delaying necessary treatment for an extended period may exacerbate the original condition and lead to further complications. As this is a preliminary study with a limited sample size, normality tests were conducted, and, when necessary, non-parametric tests were used to analyze the results. However, the findings should be interpreted with caution.

Distalizing only the first maxillary molars is generally considered a relatively straightforward procedure, particularly when using conventional distalization methods or non-compliance appliances such as headgear alternatives, distal jet, or pendulum devices. These techniques often achieve efficient movement of the first molars due to their isolated focus and the favorable biomechanics involved [10]. However, attempting to distalize both the first and second maxillary molars simultaneously presents a significantly greater challenge. The presence of the second molars increases the anchorage demand and resistance to movement, often resulting in reduced efficiency and increased treatment time. In addition, conventional and non-compliance appliances may struggle to generate sufficient and controlled distal forces to move both molars effectively without causing unwanted side effects such as anchorage loss or tipping. Consequently, simultaneous distalization of first and second molars requires more complex mechanics, precise control, and, often, supplementary anchorage strategies to achieve predictable outcomes [14].

Actually, in adult patients with fully erupted maxillary second molars, the amda^®^ can serve as a reliable alternative appliance. Skeletal anchorage enhances overall treatment control and predictability while reducing the need for patient compliance.

## 5. Conclusions

The amda^®^ seems to provide an ideal option for treating patients with Class II malocclusion, achieving an almost bodily maxillary molar distalization without other side effects of the conventional non-compliance distalization appliances.

## Figures and Tables

**Table 1:** Cephalometric variables for dentoalveolar angular and linear measurements.

Cephalometric Variables
Skeletal angular measurements (sagittal) SNA (Sella–Nasion-A point) (°) SNB (Sella–Nasion-B point) (°) ANB (A point–Nasion-B point) (°)
Skeletal angular measurements (vertical) SN- palatal plane (Sella–Nasion and Palatal Plane) (°) SN- mandibular plane (Sella–Nasion and Mandibualr Plane) (°) SN- occlusal plane (Sella–Nasion and Occlusal Plane) (°)
Dentoalveolar angular measurements SN-6 (Sella–Nasion and Maxillary First Molar) (°) SN-1 (Sella–Nasion and Maxillary Incisor) (°)
Dentoalveolar linear measurements (sagittal) PTV-6 centroid (Pterygomaxillary Vertical and Maxillary First Molar) (mm) PTV-1 (Pterygomaxillary Vertical and Maxillary Incisor) (mm) Overjet (mm)
Dentoalveolar linear measurements (vertical) Palatal plane-6 centroid (mm) Palatal plane-1 (mm) Overbite (mm)

**Table 2:** Tests for differences in cephalometric measurements between timepoints.

Measurement	Difference (Mean ± sd)	Difference [Median (IQR)]	Shapiro’s	Paired *t*-Test	Wilcoxon Signed-Rank	Bonferroni-Adjusted Significance (α = 0.004)
SNA	1.12 ± 1.89	0.00 (0.00–1.50)	<0.001	-	0.181	-
SNB	0.88 ± 1.12	0.50 (0.00–1.25)	0.036	-	0.098	-
ANB	0.88 ± 0.99	0.50 (0.00–2.00)	0.006	-	0.089	-
SN-Palatal	1.12 ± 0.99	1.00 (0.75–1.25)	0.156	0.041 *		No
SN-Mandibular	1.38 ± 1.89	1.00 (1.00–1.25)	0.010	-	0.018 *	No
SN-Occlusal	1.88 ± 0.05	2.00 (0.00–3.25)	0.094	0.120	0.057	-
SN-1	4.88 ± 4.05	4.50 (2.50–4.25)	0.532	0.011 *		No
PTV-1	2.38 ± 1.19	2.00 (1.75–3.25)	0.168	<0.001 *		**Yes**
Overjet	1.00 ± 0.92	1.00 (0.00–2.00)	0.030	**-**	**0.0498**	No
Palatal Plane-1	4.75 ± 4.16	4.00 (2.50–5.50)	0.152	0.015 *		No
Overbite	0.75 ± 0.70	1.00 (0.00–1.00)	0.055	0.020 *		No
SN-6	1.69 ± 2.68	0.75 (0.48–1.20)	<0.001	-	0.008 **	No
PTV-6	4.20 ± 1.37	4.08 (3.58–5.20)	0.834	<0.001 *		**Yes**
Palatal Plane-6	1.74 ± 1.94	1.36 (0.55–2.20)	0.039	-	0.022 *	No

**Table 3:** Tests for differences in superimposition measurements between timepoints.

Measurement	Difference (Mean ± sd)	Shapiro’s	Paired *t*-Test	Bonferroni-Adjusted Significance (α = 0.013)
Bjork sum	−0.09 ± 1.67	0.754	0.885	-
Pog to N-perp	0.44 ± 2.86	0.521	0.675	-
A to N-perp	0.37 ± 1.95	0.081	0.606	-
B to N-perp	0.41 ± 2.2	0.351	0.611	-

## Data Availability

All data generated or analyzed during this study are included in this published article.

## Supplementary Materials

The following supporting information can be downloaded at: https://www.mdpi.com/article/10.3390/jcm14176323/s1, Table S1. Test for differences in cephalometric measurements between timepoints.
