# Supplementary material for: Evaluation of Maxillary Molar Distalization Supported by Mini-Implants with the Advanced Molar Distalization Appliance (amda®): Preliminary Results of a Prospective Clinical Trial
Source: J Clin Med. 2025 Sep 7;14(17):6323. doi: 10.3390/jcm14176323 (PMC12429304; doi:10.3390/jcm14176323)
Supplement: Supplementary file 1 [file jcm-14-06323-s001.zip › jcm-3721763-supplementary.pdf]

Supplementary Table S1. Test for differences in cephalometric measurements between timepoints

**Measurement**	**Difference [median (IQR)]**	**Wilcoxon signed-rank**	**Bonferroni-adjusted significance (α=0.004)**
SNA	0.00 (0.00 1.50)	0.181	-
SNB	0.50 (0.00 – 1.25)	0.098	-
ANB	0.50 (0.00 – 2.00)	0.089	-
SN-Palatal	1.00 (0.75 – 1.25)	**0.031***	No
SN-Mandibular	1.00 (1.00 – 1.25)	**0.018***	No
SN-Occlusal	2.00 (0.00 – 3.25)	0.057	-
SN-1	4.50 (2.50 – 4.25)	**0.022***	No
PTV-1	2.00 (1.75 – 3.25)	**0.014***	No
Overjet	1.00 (0.00 – 2.00)	0.050	No
Palatal Plane-1	4.00 (2.50 – 5.50)	**0.022***	No
Overbite	1.00 (0.00 – 1.00)	**0.048***	No
SN-6	0.75 (0.48 – 1.20)	**0.008****	No
PTV-6	4.08 (3.58 – 5.20)	**0.008****	No
Palatal Plane-6	1.36 (0.55 – 2.20)	**0.022***	No
